# Targeting Human Papillomavirus-Associated Cancer by Oncoprotein-Specific Recombinant Antibodies

**DOI:** 10.3390/ijms22179143

**Published:** 2021-08-24

**Authors:** Maria Gabriella Donà, Paola Di Bonito, Maria Vincenza Chiantore, Carla Amici, Luisa Accardi

**Affiliations:** 1 STI/HIV Unit, Istituto Dermatologico San Gallicano IRCCS, 00144 Rome, Italy; mariagabriella.dona@ifo.gov.it; 2 Department of Infectious Diseases, Istituto Superiore di Sanità, 00161 Rome, Italy; paola.dibonito@iss.it (P.D.B.); mariavincenza.chiantore@iss.it (M.V.C.); 3 Department of Biology, University of Rome Tor Vergata, 00133 Rome, Italy; carami371@gmail.com

**Keywords:** antibody therapeutics, recombinant antibodies, intracellular antibodies, single-chain antibody fragment, nanobody, Human papillomaviruses, HPV oncoproteins, HPV-associated cancer, HPV cancer therapy

## Abstract

In recent decades, recombinant antibodies against specific antigens have shown great promise for the therapy of infectious diseases and cancer. Human papillomaviruses (HPVs) are involved in the development of around 5% of all human cancers and HPV16 is the high-risk genotype with the highest prevalence worldwide, playing a dominant role in all HPV-associated cancers. Here, we describe the main biological activities of the HPV16 E6, E7, and E5 oncoproteins, which are involved in the subversion of important regulatory pathways directly associated with all known hallmarks of cancer. We then review the state of art of the recombinant antibodies targeted to HPV oncoproteins developed so far in different formats, and outline their mechanisms of action. We describe the advantages of a possible antibody-based therapy against the HPV-associated lesions and discuss the critical issue of delivery to tumour cells, which must be addressed in order to achieve the desired translation of the antibodies from the laboratory to the clinic.

## 1. Introduction

In the last decades, because of the huge advances of recombinant DNA technology, recombinant antibodies have found increasing applications in the therapy of many diseases, whether of genetic, infectious, or tumour origin. Several antibodies and antibody-based products are either approved or under investigation in clinical trials and, particularly for tumours, many of them have revolutionized classical chemotherapy based on drugs [1]. Through recombinant antibodies, it is possible to interfere with specific protein functions at DNA, RNA, or protein level. Direct targeting of pathogenic proteins can even be advantageous over the targeting of genomic sequences with an on/off mode, because it allows modulating and tailoring protein activity without affecting genomic sequences.

Currently, thanks to the ability of the mammalian immune system to produce antibodies against virtually any antigen, and to over 30 years of molecular technology studies on antibody manipulation, well-established methods allow the selection of ligands for specific protein epitopes in either intra- or extra-cellular environment. Antibody selection can be performed from recombinant antibody libraries of different kinds, even originating from animals immunized with antigens of interest. Specific antibodies can be delivered directly to the cells as purified proteins or expressed as intracellular antibodies (intrabodies) by recombinant DNA technology. Different antibody formats representing more or less extended regions of an immunoglobulin (Ig) are presently available. The small size formats, i.e., antibodies in single-chain format (scFvs) and single domain antibody or nanobodies (sdAb or Nbs) [2], are the most suitable for expression as intrabodies because they are easily engineerable.

Several monoclonal antibodies (mAbs) in different formats reached the clinical stage or are in different clinical trial stages for the treatment of numerous pathologies including tumours [1,3]. We are principally interested in tumours associated to Human Papillomaviruses (HPVs), which represent a global health problem in terms of morbidity and mortality and for which many therapeutic strategies are under study. Among these, the approach based on recombinant antibodies deserves particular attention because of its potentialities related to safety, precision, and feasibility [3,4,5].

Here we describe, to the best of our knowledge, the different formats of recombinant antibodies against the HPV oncoproteins of Human Papillomaviruses characterized to date or currently under study and discuss whether and why they show promise for the treatment of pre-neoplastic and neoplastic lesions caused by these viruses.

## 2. Different Antibody Formats: mAbs, scFvs and Nanobodies

Recombinant antibody technology has undergone tremendous development in recent decades, so to prompt much progress in disease diagnosis and therapy. The use of display technologies allows in vitro selection from non-animal-derived recombinant (naïve or synthetic) repertoires (libraries) of peptides and antibody fragments in different formats such as Fab fragments (Fabs), scFvs, and Nbs. Different platforms are available such as phage display, yeast display, ribosome display, bacterial display, mammalian cell surface display, mRNA display, and DNA display. All of them mimic what occurs in vivo during antibody generation by the immune system as they rely on (1) genotypic diversity, which can be obtained by immune stimulation of a competent organism or by cloning; (2) the link existing between the genotype and phenotype; (3) selective pressure for increasing antibody specificity; and (4) amplification of specific clones originated by selective pressure. The coding sequences of binders specific for a given antigen, identified by the display technology of choice, can be expressed in prokaryotic or eukaryotic systems and tested both in vitro and in vivo for their ability to counteract the target antigen activity.

The possibility to engineer the originally identified antibody sequence represents an added value, since affinity, stability, and expression level can be improved while maintaining the desired antigen-binding properties. Furthermore, it is possible to modify the format so that the antibody could acquire new kinetic properties. Importantly, it is feasible to bypass the risk of immune reactions during clinical use by constructing antibodies from human scaffolds.

**Figure 1:**
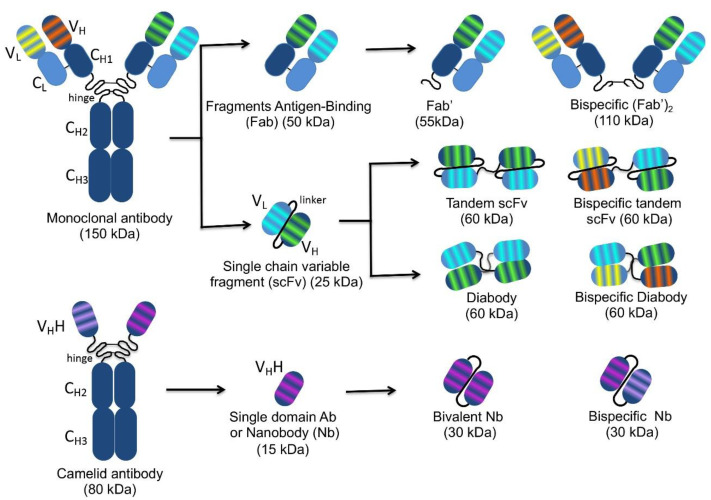
Schematic representation of the structure of conventional and camelidae monoclonal antibodies and of different antibody fragments. On the left, the whole monoclonal antibody (**top**) and camelid antibody (**bottom**) structures are represented. The variable light (VL) and heavy (VH) chains, as well as the constant light (CL) and heavy (CH1, CH2, CH3) chains are indicated. The complementarity determining regions responsible for antigen binding, three for each VL and each VH, are represented by stripes highlighted in different colours according to the different antigen specificity. Arrows indicate different monospecific and bispecific antibody fragments derived from the original antibody molecules with their nomenclature and molecular weight. The VH and VL in the different arrangements are connected by peptide linkers of 3–12 amino acids represented by black curved lines.

A whole IgG molecule (150 kDa) comprises heavy (H) and light (L) chains each consisting of a variable (VH and VL) and a constant (CH and CL) region covalently linked to each other and to oligosaccharides necessary for antibody effector functions and for long serum half-life. The antigen-binding regions responsible for diversity among antibodies are the complementary determining regions (CDRs), three for each VH and VL. The VH and VL joined by a disulphide bond and covalently linked to the first CH domain are obtainable by IgG proteolysis, resulting in a Fab monovalent antibody fragment (55 kDa) (Figure 1).

A few decades ago, it was observed that the N-terminal IgG fragment including the VH and VL retains the same antigen-binding capacity as the whole IgG molecule. The so-called scFvs (27 kDa) lack the constant regions and include only the VH and VL linked by a short peptide consisting of a sequence of glycine and serine residues such as (Gly4Ser)3. This arrangement provides flexibility, hydrophilicity, and resistance to proteases digestion. The linker length can be modified to favour or not the formation of multimers. In fact, shortening the linker to 3–12 amino acids prevents the formation of monomeric forms supporting inter-molecular VH-VL combinations also in different orientations, with spontaneous formation of a scFv dimer called “diabody” (60 kDa), where each of the two antigen-binding sites are formed by the VH of one scFv and the VL of the other one (Figure 1). The linkage of two scFvs in a unique molecule forms a tandem scFv. Both diabodies and tandem scFvs can have two different binding specificities, and in this case, they are called bispecific (bs). Interestingly, even the VH and VL arrangement in the scFv fusion protein can influence the binding activity, and it is currently possible to predict the best functional structure so as to design scFvs that meet all requirements by molecular modelling using a computer-aided homology method [6,7].

The CDRs of a scFv are embedded in an amino acid scaffold of either human or animal origin, according to the library utilized for selection. Of note, CDRs with specific binding activities can be isolated and grafted onto different scaffolds suitable for the purposes of interest. Both scFv and Fab fragments can be engineered into stable oligomers to increase binding avidity and widen antigen specificity. Specific applications of these formats are the recruitment of T-cells to tumours in immunotherapy, viral retargeting in gene therapy, and targeting of multiple antigens for a synergic/additive effect. All the mentioned antibody formats, having a size of 15–80 kDa (Figure 1), show an easy tumour penetration and are cleared from the bloodstream flow more quickly with respect to the full size IgGs (150 kDa, Figure 1). Furthermore, their genes can be easily manipulated to modify their stability, specificity, and affinity for the antigens.

Antibody engineering also allows cloning of scFv sequences into eukaryotic vectors equipped with intracellular localization signals, for the scFv expression in specific cell compartments as intrabodies. These can reach and recognize target antigens in the cellular compartments where they are located, with outcomes ranging from direct antigen blockade to indirect impairment of its activity through delocalization, to its targeted degradation [8,9].

The discovery of the smallest format of antibody fragments, the Nbs, expanded the possibilities of targeting intracellular antigens through biotechnology. Nbs derive from Camelidae species (e.g., llamas, dromedaries and camels), which in their antibody repertoire have IgG lacking both light chains and CH1 domains (heavy-chain-only antibodies: HCAbs) [10]. The variable domains of these HCAbs are named VHHs and can be isolated as single-domain antibodies (sdAbs), which are small-sized (~15 kDa) but retain the antigen-binding capability of the full-size antibody. Thanks to the small size, VHHs can easily penetrate tissues and access cryptic epitopes [11,12]. They are more soluble and capable of efficient folding with respect to conventional mAbs, which renders them suitable for high-yield production in *E. coli* and even for delivery to or expression in infected cells as intrabodies. Nbs can resist a wide pH range and high temperatures, and some of them tolerate the presence of organic compounds. Despite the non-human origin, VHHs are rarely immunogenic due to the small size and high sequence homology to the human VH3 gene family, which avoids the necessity of humanization for translation into clinic [13]. The small size also favours rapid renal clearance and facilitates the in vivo application in diagnosis rather than therapy, as the latter use requires prolongation of their half-life, which is approximately 2 h. Nevertheless, VHHs targeting haematological, oncological, infectious, inflammatory/auto-immune, bone and neurological diseases are already being evaluated in clinical trials, while the humanized VHH Caplacizumab (CabliviTM) was recently approved in Europe and USA for the treatment of acquired thrombotic thrombocytopenic purpura [14,15,16].

## 3. HPV-Associated Lesions and Current Therapies

HPVs are small, non-enveloped viruses, with a circular dsDNA genome of about 8 Kbp. The icosahedral capsid is composed of the major L1 protein and the minor L2 protein. Following infection of the epithelium basal cells, the E1 and E2 early proteins play key roles in viral DNA replication and amplification, as well as in regulating viral transcription. When actively expressed, the viral genome is maintained as an episome. Viral genome amplification, late gene expression, and viral progeny assembly are limited to terminally differentiated layers of the epithelium. Since terminally differentiated keratinocytes undergo growth arrest, HPV genomes have evolved, as a replication strategy, the expression of E6, E7, and E5 proteins, which are able to keep the infected cells in a competent state for DNA synthesis. Most HPV infections usually clear within 1–2 years but, in some cases, the virus may persist and occasional integration of its genome in the host’s genome may occur. Consequently, the infected cells overexpress E6 and E7 due to the loss of the E2 transcriptional repressor coding sequence.

HPV infections are among the most common sexually-transmitted diseases and cause annually around 5% of all cancers worldwide [17,18,19]. However, only some genotypes are responsible for morbidity and mortality related to cancer, mostly of the cervix (CC) but also of the anogenital area and of the oropharynx, whose number is constantly increasing. According to the different oncogenic potential, HPVs are defined as high-risk (HR) types, which may cause the development of high-grade squamous intraepithelial (H-SIL) and cancer lesions, and low risk (LR) types, mainly causing anogenital warts. Worldwide, the HR HPV16 and HPV18 cause 71% of CC, with the remaining genotypes causing the residual HPV-associated cases [20,21].

Since more than 10 years, a bivalent vaccine against HPV16 and HPV18, and a quadrivalent vaccine that also targets the LR HPV6 and HPV11, responsible for genital warts, have been available. Recently, a nonvalent vaccine that offers protection against the five other most common HR genotypes (HPV 31, 33, 45, 52, and 58) and prevents about 90% of CC cases was developed [22]. The vaccines consist of DNA-free virus-like particles (VLPs) obtained by the only expression of the viral L1 protein through recombinant technology, and are administered with proper adjuvants. Nevertheless, effective prevention of the HPV-associated pathologies is expected only in the long-term if HPV vaccination is able to reach a significant percentage of the target population worldwide [22,23,24]. The three HPV vaccines (bivalent, tetravalent, and nonavalent) have been licensed as prophylactic vaccines, but currently only 8% of low- and middle-income nations have introduced HPV vaccination programs in their health policies. In addition, in recent years many trials were performed to investigate the efficacy of these vaccines in preventing HPV disease recurrence after the treatment of high-grade cervical or anal lesions and condylomatosis, showing some promising results [24].

Of note, the 73rd World Health Assembly, held on August 2020, strongly encouraged the acceleration of actions aimed at eliminating cervical cancer as a public health problem. This should be pursued through “the inclusion of HPV vaccine into national immunization programs” and the “improvement of availability, affordability, accessibility, utilization and quality of screening, vaccines, diagnostics, and treatment and care of pre- and invasive cervical cancer” [23].

Treatment of HPV-associated lesions varies widely, mainly depending on lesion grade (high-grade lesions versus invasive cancer), lesion localization (lower genital tract, uterine cervix or head and neck), and tumour stage. In general, current therapeutic approaches aim at eliminating abnormal/malignant cells. This can mainly be achieved through surgery, chemotherapy, radiotherapy, targeted therapy, or immunotherapy. These approaches can be used alone or, in some cases, in combination.

In HPV-associated malignancies, E6 and E7 oncoproteins represent tumour-associated antigens, which are ideal targets for the development of vaccines stimulating specific cytotoxic T lymphocytes (CTLs). In HPV natural infection, E6- and E7-specific CD4 and CD8 immune responses are associated with the regression of HPV-cervical lesions. However, therapeutic vaccines are not yet available for clinical practice in spite of the numerous clinical experimentations carried out during the last 25 years, which used E6 and E7 through different platforms to raise specific immunity. The poor efficacy of these vaccines, due the lack of specific adjuvants of T-cell responses, has led to the interruption of many clinical trials in the early stages. Only recently, an improvement in efficacy was achieved by combining E6-E7 therapeutic vaccines with the use of radio- and chemotherapy (Cisplatin, Carboplatin, Paclitaxel), immune check-point inhibitors (anti-PD-1, anti-PDL1, anti-CTL4), cytokines (IFNalpha and IL-12), angiogenesis inhibitors (anti-VEGF), and modulators of tumour microenvironment (TME, Histone Deacetylase Inhibitors). Several new clinical trials are currently underway and the time for any authorizations has been further lengthened [24,25].

This context accounts for the huge bulk of research, particularly in the field of immunotherapy, still underway on therapeutic approaches against HPV lesions [26]. One of the promising treatments is undoubtedly that involving the use of intrabodies to block viral oncoproteins activities, and will be described below.

## 4. Molecular Mechanisms of HPV-Induced Carcinogenesis

HPV-associated carcinogenesis is known to be driven by the expression, in the transcription order, of the non-structural E6, E7, and E5 oncoproteins encoded by early genes [27].

**Figure 2:**
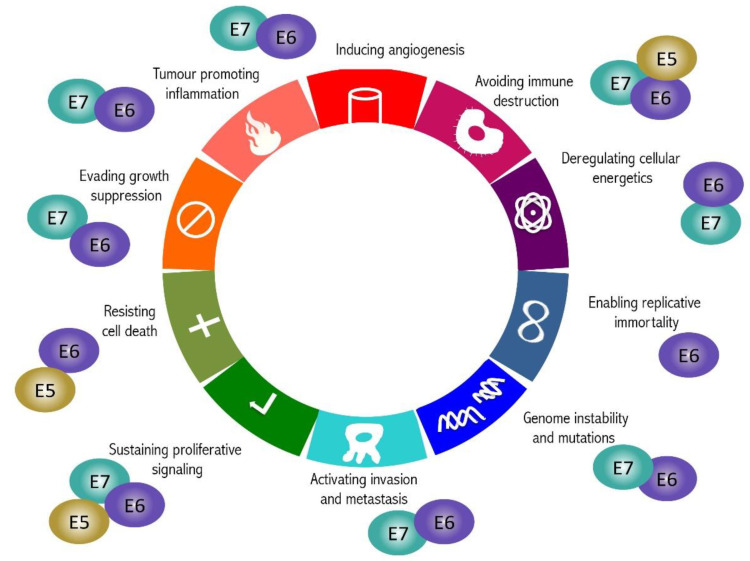
Involvement of HPV oncoproteins in cancer hallmarks. Dysregulation of cell pathways ascribable to E6, E7, and E5 oncoproteins of HR HPVs is responsible for the entire spectrum of hallmarks of Human Papillomavirus (HPV)-related cancer (see text for details). In the figure, the involvement of each oncoprotein in the different hallmarks is reported.

The activity of E5, E6, and E7 has not been conclusively characterized and new functions are constantly being discovered. The following sections will briefly describe those molecular targets and biological activities that, causing the subversion of important regulatory pathways, are associated with the hallmarks of cancer as outlined by Hanahan and Weinberg [28]. In some instances, the role of the oncoproteins in determining a specific hallmark is well characterized, while in other cases, the precise activity connected with a hallmark has yet to be fully elucidated and the available lines of evidence provide only hints on the link between HPV oncoproteins and some hallmarks. A schematic representation of the HPV oncoprotein role in determining the hallmarks of cancer is shown in Figure 2.

### 4.1. Cell Cycle Deregulation and Sustaining of Proliferative Signalling

Deregulation of cell proliferation is one of the most characteristic traits of malignant cells. E7 oncoprotein targets key cellular mediators leading to uncontrolled cell proliferation. In particular, it establishes well-characterized interactions with the members of the “pocket proteins” family, represented by pRB, p107, and p130, and promotes their degradation [29]. This, in turns, allows the release of the E2F transcription factor, with the consequent expression of S-phase genes as well as of the p16INK4A cyclin-dependent kinase inhibitor. S-phase entry is also induced by E7 through the direct inactivation of essential regulators of G1-to-S-phase transition, such as p21 and p27 cyclin inhibitors. Sustained proliferation of HPV-infected cells is also induced by E5 oncoprotein, which increases the Epidermal Growth Factor Receptor (EGFR) signalling activity by forming an activating complex with it and impairing its degradation [30].

### 4.2. Resistance to Cell Death

Escape from cell death is one of the most important hallmarks of cancer, allowing cells with genomic defects to survive and continue to proliferate [31]. E6 interferes with several cell death pathways. It can abrogate apoptosis by promoting proteasomal degradation of p53 tumour suppressor, which controls the expression of pro-apoptotic genes [32]. E6 further promotes cell survival by impairing cell response to tumour necrosis factor (TNF), protecting the infected cells from Fas-induced apoptosis [33] and transactivating survivin gene promoter [34]. E5 oncoprotein also supports antiapoptotic activity during the first steps of the oncogenic process through downregulation of Fas-Ligand (Fas-L) on the cell membrane [33] and increased degradation of Bax [35].

### 4.3. Avoiding Immune Destruction

HPVs have developed mechanisms to counter the destruction of infected cells by the host’s immune system. In this context, E5 plays an important role by promoting MHC class I retention in the Golgi apparatus, resulting in impaired viral antigen recognition [36]. Recently, E5 was found to be also involved in the increased expression of PD-L1 and inhibition of effector T cells, events that facilitate the immune evasion of HPV-infected cells [37]. E6 plays a direct role in modulating the immune response against HPV antigens by impairing Interferon (IFN)-mediated host defence in different and interrelated ways [38,39]. E7 also interferes with IFN signalling [40] and inhibits the Toll-like receptor-9 (TLR9) recognition in cooperation with E6 [41].

### 4.4. Replicative Immortality

To ensure the unlimited cell proliferation associated with carcinogenesis, E6 induces overexpression of human telomerase reverse transcriptase (hTERT), the catalytic unit of the human telomerase [42]. This occurs through both direct promoter activation and proteasomal degradation of its transcriptional repressor NFX1-91 through the E6/E6AP complex [43]. Constitutive expression of hTERT is also established through epigenetic mechanisms depending on alteration of the activity of histone methylases and demethylases [44], with E6 representing the main player.

### 4.5. Induction of Angiogenesis

HPV-infected cells derive sustenance and oxygen from the surrounding tissues thanks to the ability of the viral oncoproteins to induce angiogenesis. E6 and E7 regulate several angiogenesis modulators, including both inducers and inhibitors of this process. Both E6 and E7 are able to trigger the angiogenic switch by upregulating the expression of Vascular Endothelial Growth Factor (VEGF) respectively through direct [45] or E2F1-mediated [46] transcriptional activation of the angiogenetic factor gene [47]. In response to E6 and E7 expression, Interleukin-8 (IL-8), a major angiogenesis inducer, is also promoted [48]. On the other hand, expression of the thrombospondin-1 and maspin inhibitors of angiogenesis is indirectly perturbed by E6 as a result of E6-mediated degradation of p53, which positively regulates these inhibitors [48].

### 4.6. Deregulation of Cellular Energetics

Alterations of cell metabolism are among the earliest changes observed in cancer cells. Both the E6 and E7 oncoproteins contribute to the switch from oxidative to glycolytic cell metabolism, known as Warburg effect. Mechanisms by which HPV oncoproteins induce reprogramming of cell metabolism are reviewed in detail elsewhere [49]. E6 interferes with the expression of genes involved in the control of glycolytic metabolism through its interactions with p53 and c-Myc, while E7 promotes the glycolytic pathway by upregulating the expression of different glycolytic enzymes, such as hexokinase, involved in the first step of glucose metabolism, and by direct binding and activation of M2-pyruvate kinase. In addition, HPV16 E5 does not directly regulate glycolytic enzymes but contributes to the metabolic switch by activating the EGFR pathway that, in turn, promotes an enhancement of the glycolytic metabolic program [49].

### 4.7. Invasiveness and Metastasis Induction

The E6 encoded by HR HPVs plays a pivotal role in this cancer hallmark as it downregulates proteins modulating cell polarity and motility such as the scribbled planar cell polarity protein (SCRIB) involved in cell polarization and differentiation, and the membrane-associated guanylate kinases 1, 2, and 3 (MAGI-1, 2 and 3) [50]. Furthermore, E6-mediated functional modulation or degradation of adhesion effectors allows matrix-independent cell growth [51], resulting in enhanced motility and invasion of HPV-positive cells. E6/E7 contribute to the metastatic and invasive behavior of HPV-positive tumours by increasing the expression of different matrix metalloproteinases (MMPs) [52]. Both E6 and E7 are involved in the epithelial–mesenchymal transition (EMT), crucial process for invasion and metastasis, by regulation of E-cadherin [53,54] and N-cadherin [55]. HPV-induced invasive cancer behavior has recently been correlated with E5 activity as well [56]. E5 is in fact able to upregulate the growth factor receptor MET, critical for tumour cell invasion, motility, and cancer metastasis, at both protein and mRNA level. Through this activity, E5 contributes to increase motility of the HPV-positive keratinocytes, and may thus promote metastasis of HPV-associated malignancies.

### 4.8. Genome Instability

The uncontrolled cell proliferation promoted by HPV oncoproteins facilitates the accumulation of genetic aberrations and genomic instability. E7 plays a central role in this process by inducing centrosome aberrations [57]. By increasing the activity of the cyclin-dependent kinase 2 (CDK2), E7 further leads to an augmented risk of genomic instability [58]. Concomitantly, a higher mutation rate is caused by E6 [59]. Altogether, E6 and E7 interfere with almost all the main actors of the cellular DNA repair pathway, thus reducing and delaying the removal of damages from the host cell genetic material. Another important hallmark associated with the expression of E6 and E7 is the interference with effectors of epigenetic modifications (e.g., DNA methyltransferases and histone modification enzymes). By influencing their activity, HPV oncoproteins may cause either the activation of oncogenes or the silencing of tumour suppressor genes [60].

### 4.9. Tumour-Promoting Inflammation

Through various mechanisms, E5, E6, and E7 are all involved in the development of chronic inflammation, a major cofactor of malignant transformation. In fact, the expression of genes involved in the inflammatory response increases due to E6/E7 activity. Persistent expression of oncoproteins leads to changes in the release of several pro-inflammatory cytokines (e.g., IL-6 and IL-18) and chemokines with a well-characterized role in inflammation and carcinogenesis. By acting in an autocrine manner, cytokines affect keratinocyte differentiation, proliferation, and secretion of other soluble mediators while, in a paracrine manner, they lead to the increase of infiltrating inflammatory cells. This tumour microenvironment contributes to tumour growth, angiogenesis, resistance to apoptosis, and local immune surveillance [61]. In addition, HPV oncoproteins stimulate cyclo-oxygenase-2 (COX-2) production with the consequent activation of the COX-prostaglandin (PG) pathway, which is considered the main cause of HPV-induced inflammation [62].

## 5. Therapeutic Recombinant Antibodies Targeting HPV Oncoproteins

Safe and non-invasive interventions without side effects would be desirable for the treatment of HPV-related lesions. In order to be effective also in immunodeficient patients, therapeutic strategies possibly not involving the individual immune response would be recommended.

An effective and timely treatment of pre-neoplastic lesions could avoid their progression toward invasive cancer. At the same time, a well-timed and effective therapy for already established tumours could ameliorate patient prognosis. Another important therapeutic area of intervention could be the prevention of metastases deriving from surgically removed HPV tumours.

Much progress has been made in the last years to develop therapeutic vaccines against HPV-associated tumours. The main platforms include peptide- and protein-based vaccines; DNA virus- and RNA virus-based vectors; bacterial vectors; cell-based, DNA-based, and RNA-based vaccines; and vaccines combining two of the mentioned platforms. Other lines of research involve the combined use of therapeutic vaccines with other treatment modalities such as PD-1/PD-L1 axis inhibitors or other checkpoint inhibitors, HDAC inhibitors or other treatments. Many clinical trials are ongoing, with some having even reached Phase II. They are reviewed in detail elsewhere [25]. Nevertheless, critical issues remain to be solved such as anti-vector immunity, HLA-restriction of peptides, or the difficulty of identifying valid outcomes to compare the efficacy of these strategies.

In view of their crucial role in the onset and progression of HPV-driven tumours [63], E5, E6, and E7 proteins represent ideal targets for alternative anti-tumour therapeutic approaches based on protein knock out or knock down methods. In this context, recombinant antibodies seem to represent a valid therapeutic opportunity. Currently, a number of recombinant antibodies against the HPV oncoproteins are available in different formats. In recent years, such antibodies have been tested for their therapeutic potential against HPV-associated disease. They are summarized here referring to their targets.

### 5.1. E6-Specific Recombinant Antibodies

Many studies demonstrated that E6 protein, with its multiple direct and indirect interactions, is an undiscussed druggable target [64]. Since its X-ray structure in complex with E6-associated protein (E6AP), the ubiquitin ligase involved in p53 polyubiquitination, and p53 has been resolved, the possibility of inhibiting this complex has been increasingly investigated through several methods among which those based on specific recombinant antibodies appear promising.

#### 5.1.1. ScFvs and mAbs

**Figure 3:**
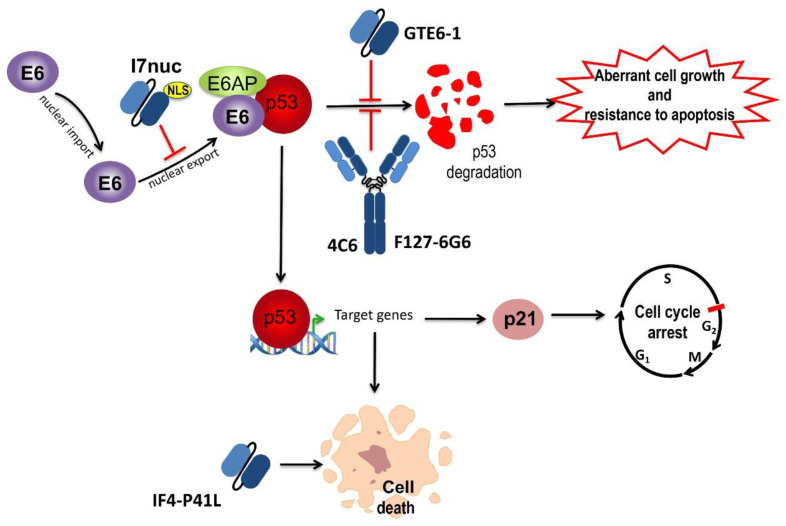
Schematic representation of the known effects of anti-E6 intrabodies expressed in HPV-positive cells. The effects of the intracellular expression of specific single-chain antibody fragments (scFvs) (GTE6-1 and IF4-P41L) and monoclonal antibodies (mAbs) (4C6 and F127-6G6) are shown. The binding of I7nuc to E6 inhibits its nuclear export and the subsequent cytoplasmic degradation of p53. Similarly, E6 binding by the GTE6-1 scFv or 4C6 and F127-6G6 mAbs inhibits p53 degradation, preventing the resistance to apoptosis and leading to the uncontrolled cell proliferation characteristic of HPV-positive cells. The rescue of nuclear p53 levels activates the transcription of genes involved in the induction of apoptosis and in the control of cell proliferation. The pro-apoptotic effect of IF4-P41L scFv, apparently p53-independent, is also shown.

The 1F1 and 6F4 (F4) scFvs, derived from mAbs obtained by mice immunization with the GST-HPV16E6 fusion protein and targeting the E6 N-terminus, were able to hamper p53 degradation in vitro by inhibiting the formation of the E6/p53 complex [65]. Lagrange et al. characterized three further anti-16E6 mAbs (1F5, 3B8, 3F8) targeting the 16ZD2 zinc-binding domain, which were able to bind E6 through a shared 16 amino acid sequence. By comparing the activity of these mAbs to that of scFv F4, they found opposite effects, with the mAbs being unable to affect the E6AP-dependent and able to affect the E6AP-independent binding of p53, possibly as a consequence of an antibody-induced conformational change at the E6AP-binding site of E6 [66]. The capacity of intracellular folding and cytosolic stability/solubility of scFvF4 was improved by mutagenesis, obtaining the IF4-P41L scFv [66]. Such scFv expressed by adenoviral system was able to cause specific apoptosis of HPV16-positive cells in a way proportional to the scFv solubility and not related to p53 rescue, showing not to depend on the block of p53 degradation [67] (Figure 3).

Courtête et al. delivered the anti-16E6 4C6 mAb to HPV16- and HPV18-positive cells and found a specific p53 accumulation in the nucleus of HPV16-positive cells, which was favoured in the presence of a network of scFv peptide dimers linked through COOH-terminal Cysteine residues (Figure 3). Interestingly, cell proliferation was hampered but apoptosis was not restored, and a synergistic effect was obtained by co-delivery of silencing RNA targeting E6 [68].

GTE6-1, a 16E6 binder selected from a scFv library constructed by Griffin et al., was able to bind to the first zinc finger of E6 with high affinity. GTE6-1 was able to recognize specifically both partially denatured and native E6 and to inhibit E6-mediated degradation of p53 in vitro [69] (Figure 3). To evaluate the capability of antibodies in different formats to hamper the E6 activity, Griffin et al. expressed the GTE6-1 scFv also as a diabody and a triabody in a number of cell lines whose proliferation depends on E6 and E7, and compared such capability to that of peptides containing the E6-binding motif ELLG. Only the scFv format induced significant nuclear apoptosis and p53 rescue in HPV16-positive cells. The reason for the poor biological effect of diabody and triabody probably relies on the size-dependent inability to diffuse through nuclear pores. The ELLG-containing peptides exhibited high target avidity but were not effective as inhibitors of E6 function.

By delivering the 16E6-targeting F127-6G6mAb to HPV16-positive cells by sonoporation, Togtema et al. were able to reduce the E6-mediated p53 degradation but not to induce apoptosis [70] (Figure 3). Nevertheless, the effect was transient probably due to the inability of molecules as large as mAbs to penetrate the cell nucleus, and the outcome was different in the two HPV16-positive cell lines utilized, suggesting that different treatment plans might be necessary for in vivo tumour therapy. No mAb effect was observed in HPV-negative cells, confirming the safety of a mAb-based treatment, effective only in tumour cells.

Direct selection of scFvs as intrabodies is appropriate to identify stable binders able to recognize intracellular antigens such as E6 and E7. Indeed, scFvs unstable in reducing the intracellular environment will spontaneously exclude themselves from selection. In our studies, we selected from the SPLINT library [71] the anti-16E6 scFvI7 intrabody by Intracellular Antibody Capture Technology (IACT), which allows performing selection in the intracellular environment. In the light of E6 activity in the cell nucleus, we provided scFvI7 with a signal for nuclear localization (NLS), and tested the I7nuc effect in HPV16-positive cells. When co-transfecting the same cells with I7nuc and the recombinant E6, we observed I7nuc/E6 co-localization in cell nucleus. I7nuc caused a partial rescue of p53, which accumulated in cell nucleus and was able to markedly hamper cell proliferation and induce apoptosis and necrosis of SiHa cells [71] (Figure 3).

**Figure 4:**
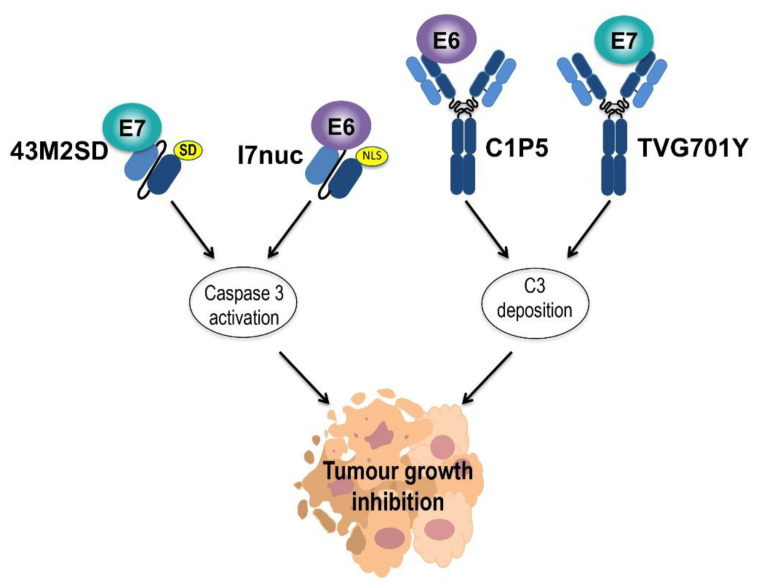
Antitumour effect of anti-E6 and anti-E7 recombinant antibodies in vivo. The effect of the anti-16E7 (43M2SD with localization in the endoplasmic reticulum, ER) and anti-16E6 scFvs (I7nuc with localization in the cell nucleus) in HPV-driven tumour mouse models is shown on the left. Mice tumours were electroporated after injection of scFv-expressing plasmids, resulting in the induction of apoptosis and large necrotic areas in the tumour mass due to caspase 3 activation. The effect of intratumour injection of anti-16E6 anti-16E7 mAbs in HPV16-positive tumour-bearing mice is shown on the right. The significant inhibition of tumour growth by C1P5 and TVG701Y might be due to the complement C3 deposition.

We then investigated the I7nuc antitumour activity in mouse models for HPV tumours based on the injection of HPV16-positive tumour cells in C57BL6 mice. The scFv capability to either prevent cancer development from scFv-expressing tumour cells, or to hinder cancer progression by delivery to already established tumours, was evaluated [71,72] (Figure 4). We observed a clear impairment of tumour growth in all mice injected with TC-1 tumour cells expressing I7nuc by retroviral transduction before inoculation into mice, with 60% of them completely protected from tumour onset for the 4 months of observation time [71]. In a different experimental setting, we delivered the scFvI7-expressing plasmids by electroporation to newly implanted tumours. Even in this case, it was possible to hamper tumour growth, providing the proof of principle for a scFv-based early treatment of cancer. The result was confirmed using two different HPV16-positive tumour cells, namely C3 and TC-1 cells, and also by employing higher amounts of TC-1 cells to mimic tumours that are more aggressive. Through histology and immunohistochemistry, we showed that the antitumour activity is based on the induction of tumour cell death by apoptosis [72].

In view of a possible direct use of antibodies in protein format for therapeutic purposes, we expressed the anti-16E6 scFv coding sequences, provided or not with the NLS, in prokaryotes. We tested the stability, reactivity, and specificity towards 16E6, of I7 and I7nuc proteins purified from *E. coli* in soluble form. The scFvs in protein format delivered to HPV16-positive cell lines were able to recognize the endogenous monomeric E6 in the cell nucleus and hampered the proliferation of these cells. These results could have interesting implications for therapy also in consideration of the high safety of scFv proteins, even though a prolonged administration over time would be necessary as the proteins are subject to degradation [73].

A recent study by Jiang et al. utilized anti-16 E6 and -16 E7 mAbs in an experimental murine model based on HPV16-positive CaSki cells implanted in Balb/c nude mice. Two different doses of the anti-16E6 C1P5 and anti-16E7 TVG701Y mAbs were delivered via intraperitoneal or intratumour injections and both showed significant ability to specifically inhibit tumour growth at an extent comparable to the Cisplatin chemotherapeutic agent. Inhibition of tumour growth was virus-specific and suggested that the mechanism underlying the mAb activity consists of a specific effect causing complement deposition and a non-specific effect on macrophage polarization [74] (Figure 4). Accumulation of complement in tumour tissue facilitates the elimination of cancer cells due to opsonisation, and significantly activates complementary pathways, thus promoting surveillance by the immune system.

#### 5.1.2. Nanobodies

Nanobodies represent the new generation of anti-HPV recombinant antibodies (Figure 1). In view of the preferential localization of 16E6 protein in the cell nucleus [75], and of their advantageous properties of thermal and chemical stability, VHHs are increasingly attracting interest for the targeting of E6. Indeed, they can penetrate into the nucleus through nuclear pores and interact with epitopes inaccessible to conventional mAbs. Three VHHs binding to the recombinant E6 with nanomolar affinities were identified from two llama, immune VHH phage display libraries by Togtema et al. [76]. The capacity of the selected VHHs to bind the native E6 derived from HPV16-positive biological samples had not yet been determined at the time of the study, nor had the bound E6 epitopes been characterized.

More recently, a different 16E6-targeting nanobody was isolated and characterized [77]. Zhang et al. advocated the possible therapeutic use of such nanobody to counteract HPV-induced tumours given its capacity to inhibit both the proliferation of HPV16 -positive cells in vitro and the growth of xenograft tumours in nude mice.

Celegato et al. explored a different approach also based on nanobodies and equally aimed at neutralizing the E6 ability to degrade p53. VHHs against the degradation-binding domain (DBD) of p53 were developed and shown to stabilise nuclear p53 in HeLa cells, which harbour the HPV18 genome, with a specific effect for HPV-positive cells. Nevertheless, the VHHs were unable to rescue the p53 tumour-suppressive functions. The authors hypothesized that this was due to inhibition of p53 transactivation associated with an increased cell proliferation and viability, and highlighted that anti-p53 DBD VHHs were able to modulate protein properties even if not reaching the desired antiproliferative effect [78].

### 5.2. E7-Specific Recombinant Antibodies

The E7 protein of HR HPVs cooperates with E6 protein to drive oncogenesis mainly through deregulation of growth suppressors, which leads to uncontrolled cell proliferation as above detailed. Therefore, E7 is widely studied as a therapeutic target, and the now in-depth knowledge of its functions suggests that specific recombinant antibodies may be a useful tool to fulfil the anticancer purposes [79].

#### 5.2.1. ScFvs and mAbs

**Figure 5:**
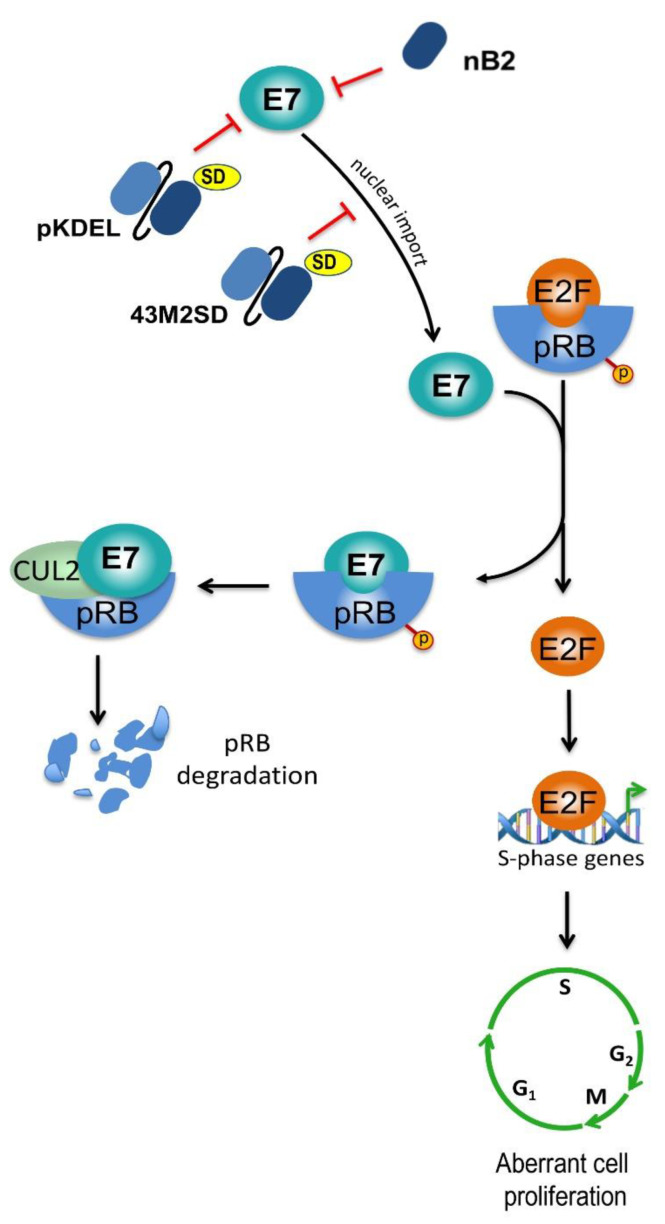
Schematic representation of the known effects of anti-E7 intrabodies expressed in HPV-positive cells. The effects of the intracellular expression of specific scFvs (pKDEL and 43M2SD) with localization in the ER are shown in the figure. pKDEL reduces the intracellular levels of E7, thus hampering its effect on cell proliferation. The binding of 43M2SD to E7 inhibits the translocation of the oncoprotein to the cell nucleus. This, in turn, hampers E7-mediated inactivation of Retinoblastoma (pRB), which regulates E2F activity on S-phase genes. The binding of 43M2SD and pKDEL can also inhibit the proteosomal pRB degradation mediated by the Cullin 2-RING ubiquitin ligase complex (CUL2). The effect of intracellular expression of the nB2 nanobody, which inhibits cell proliferation with a mechanism not yet investigated, is also shown in the figure.

The first scFvs selected against 16E7 oncoprotein were constructed directly from murine spleen cells, and then provided with signals for subcellular localization by cloning [80]. When the scFv-expressing plasmids were transfected in HPV16-positive cells, the scFv with SEKDEL signal for localization in the endoplasmic reticulum (ER) was effective in decreasing E7 expression in a manner inversely related to the amount of plasmid used for cell transfection. Interestingly, when trying to generate cells stably expressing the anti-E7 scFvs, the researchers observed that stable expression of these antibodies was not compatible with clonal outgrowth of E7-expressing tumour cells. In fact, the expression of anti-16E7 scFvs, and of that with localization in the ER in particular, successfully and specifically inhibited the proliferation of HPV16-positive CaSki and SiHa cells (Figure 5). Wang-Johanning et al. concluded that the alteration obtained was due to the interaction between the scFv and E7 [80].

More recently, our group selected from a Phage library of human recombinant antibodies, three different scFvs against the 16E7, provided them with signals for localization in the cell nucleus or ER by cloning in eukaryotic vectors, and evidenced their specific and significant antiproliferative effect in HPV16-positive cells in vitro [81]. We also characterized the scFv-binding regions by epitope mapping using immunoassays based on GST-tagged E7 proteins carrying deletions or aminoacid variations. This allowed deciphering E7 regions targeted by scFvs, and revealed that different regions known to be directly involved in transforming activities of E7 are bound by the scFvs. This suggested that different scFvs may be used to target diverse E7 activities [82,83]. We were able to improve the half-life and thermal stability of the most reactive of the anti-16E7 scFvs, scFv43, by site-directed mutagenesis, confirming that small variations in the amino acid sequence can modify the antibody biophysical characteristics [84]. We then provided the modified scFv, namely scFv43M2, with the SEKDEL signal (SD) for localization in the ER and thereafter tested the resulting scFv43M2SD for its ability to counteract the 16E7 activity. In SiHa cells, the intrabody was able to subtract E7 from the usual localization and cause it to accumulate in the ER. In addition, the scFv43M2SD intracellular expression was able to inhibit significantly and specifically the proliferation of different HPV16-positive cell lines [85]. The scFv43M2 was then tested in vivo in mouse HPV tumour models, demonstrating the ability to counteract tumour progression both when administered to tumour cells before their injection into mice and when administered to already implanted tumours [72,85] (Figure 4).

The anti-tumour activity of the anti-16E7 TVG701Y mAb has been described together with the anti-16E6 C1P5 mAb in the previous paragraph, as the two mAbs were utilized in the same study [74] (Figure 4).

#### 5.2.2. Nanobodies

Li et al. selected four VHHs with high affinity for 16E7 from llama libraries by Phage display, and one of these was chosen for further analyses because of lacking Cysteine residues potentially able to form intra-molecular disulphide bonds. The nanobody was expressed in prokaryotic system as a protein, and its ability to bind to the recombinant E7 in vitro was confirmed in immunological assays. Furthermore, the nanobody was able to detect the endogenous E7 protein in Western blotting and, most importantly, induced a specific inhibition of the proliferation of HPV16-positive cells when these cells were transfected with a recombinant eukaryotic plasmid [86] (Figure 5).

### 5.3. E5-Specific scFvs

**Figure 6:**
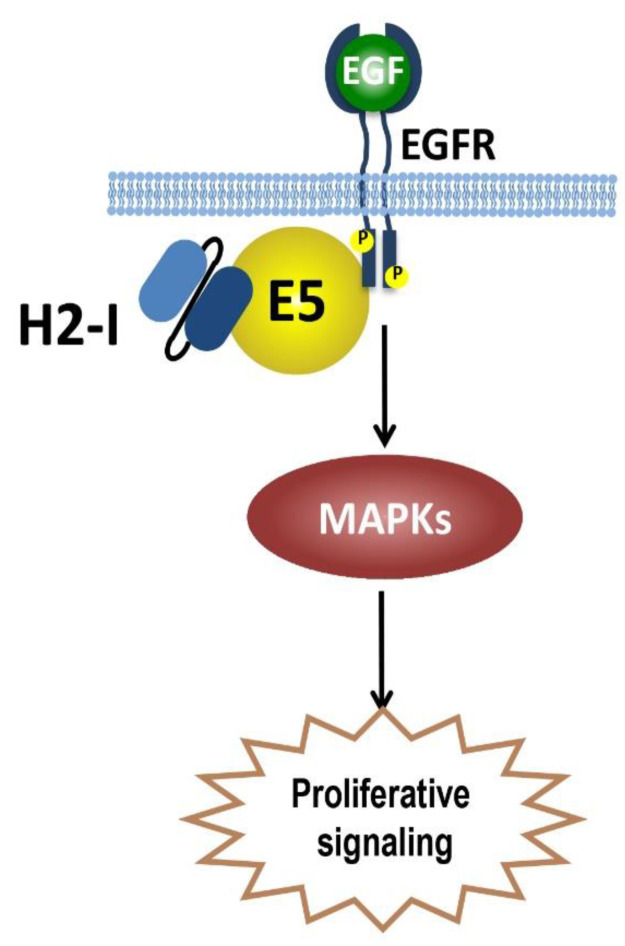
Representation of binding of the H2-I intrabody to E5. The scFv H2-I, when expressed within HPV-positive cells, colocalizes with E5 and its target, the Epidermal Growth Factor Receptor (EGFR), able to activate the mitogen-activated protein kinase (MAPK) signalling cascade, leading to DNA synthesis and cell proliferation.

In light of the recognized tumourigenic role in the early phases of HPV-induced carcinogenesis and in immunoevasion, E5 protein can be also considered a suitable target for therapeutic purposes, possibly in combination with the main E6 and E7 oncoproteins. Nevertheless, the first and currently only scFv anti-HPV16 E5 (16E5) was developed with the purpose of investigating the E5 functions [87]. Monjarás-Ávila et al. selected this antibody by Phage display technology against the recombinant 16E5 fused to Maltose-binding protein to bypass difficulties due to the E5 hydrophobicity (Figure 6). They then tested this E5-specific scFv in W12 cells, with immortalized keratinocytes carrying up to a maximum of 1000 episomal copies of the HPV16 genome at a low number of passages [88]. The scFv was able to recognize E5 in W12 cells and to reveal its co-localization with EGFR. Therefore, it deserves further investigations to explore its possible application in the therapeutic field.

### 5.4. E6 and E7-Specific Affibodies

Although they are not properly recombinant antibodies, a mention is deserved by affibodies, a new class of single-domain protein scaffolds based on non-Ig Z domain derived from the staphylococcal protein A. Affibodies are very small molecules (6 kDa) that can be selected against any protein target, and are attracting the attention of the scientific community for biotechnological applications, in particular for in vivo imaging but also for anticancer therapy. Some anti-16E6, -16E7, -18E6, and -18-E7 affibodies were selected and tested successfully both in diagnostic and therapeutic applications [89,90,91], either as bs affibodies [92] or as fusion with toxins (affitoxins) [93,94].

## 6. Intracellular Delivery Methods for Recombinant Antibodies against HPV Oncoproteins

**Figure 7:**
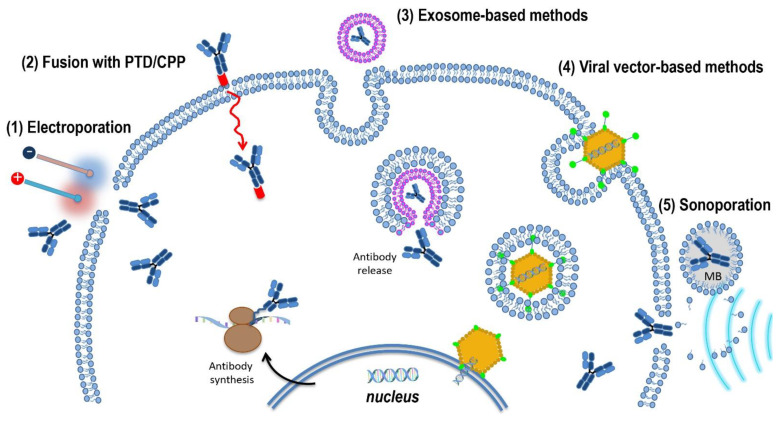
Schematic representation of delivery systems for recombinant antibodies. Some delivery systems already in use or potentially usable for the delivery to cells of mAbs and antibody fragments are illustrated. The mechanisms of cell entry are schematized for: (1) Electroporation; (2) Fusion with protein transduction domains (PTD)/Cell-penetrating peptides (CPP), shown with a red tail; (3) Exosome-based methods (entry by endocytosis is depicted as an example); (4) Viral vector-based methods; and (5). Ultrasound-based methods (sonoporation). MB, microbubbles.

In the various experimental contexts described above, recombinant antibodies showed to be effective in hindering the action of the HPV E6 and E7 oncoproteins, thus interfering with the main cancer hallmarks in which they are involved. Despite the safety and benefits of what would be a recombinant antibody-based therapy for HPV-associated lesions, still a low number of Nbs, scFvs, and mAbs against HPV oncoproteins have been developed, and none of them has reached the clinical stage so far. One of the reasons behind this essentially lies in the difficulty of identifying a delivery method that allows recombinant antibodies to cross biological barriers while maintaining biological activity, particularly when the targets are intracellular. In fact, when the target antigens are on the cell plasma membrane, the therapeutic antibodies diffuse in the extracellular environment from the bloodstream to the body tissues until they reach the target. In the case of intracellular targets, the delivery must be made first to the tumour cells, and secondly to the intracellular environment. Several studies are underway to address this criticality and permit translation to humans. In general, recombinant antibodies for intracellular targets can be either expressed within cells from DNA plasmids or delivered directly to cells as purified proteins. This is achievable by physical methods, transfection, electroporation, or fusion with a peptide transduction domain (PTD) or nanocarriers. Delivery as proteins guarantees high safety but implies the need for large quantities of Good Manufacturing Practices (GMP)-grade purified products. Without wishing to be exhaustive since the topic of “delivery” is addressed in more detail elsewhere [95], here we will mention potentially useful methods for the in vivo delivery of therapeutic antibodies against HPV E6, E7, and E5, some of which implement or are alternatives to those already explored for in vitro and in vivo use (Figure 7).

### 6.1. Electrotransfer/Electroporation

EP applies voltage pulses to generate an electric field between two electrodes, which interrupts the integrity of cell membranes with the formation of pores allowing cell uptake of nucleic acids as well as proteins. EP is therefore a safe method for intracellular protein expression since it avoids insertional mutagenesis and immunogenicity problems inherent in other methods. As such, it can be exploited in a wide range of applications, particularly in immunotherapy [96]. One of the studies reported here used EP to achieve efficient expression of therapeutic scFvs injected as DNA plasmids in HPV-driven tumours [72]. Nevertheless, the methodology could even be used to deliver scFvs as proteins or mRNAs. Indeed, RNA electroporation of hematopoietic cells has been used successfully for two decades [97].

### 6.2. Fusion with Protein Transduction Domain

PTDs or cell penetrating peptides (CPPs) are cationic and/or hydrophobic 10–30 amino acid long peptides that can be conjugated or fused to antibodies to make them able to penetrate the cell membrane via different mechanisms [98]. However, for effective translation in the clinic, the CPP-based delivery has some limitations to circumvent, mainly due to low in vivo stability and reduced binding capability.

### 6.3. Exosome-Based Methods

In our laboratories, an exosome-based strategy was recently investigated in vitro for the delivery of one anti-16E7 scFv previously studied, showing promise for translation to humans [99]. The approach relies on the property of a functional defective Nef protein of HIV-1 (Nefmut), acting as an exosome-anchoring protein for proteins fused to its C-terminus. The scFv43M2 delivered to HPV16-positive cells by engineered extracellular vesicles (EVs) carrying the Nefmut/43M2s chimeric product, was able to reproduce the already observed antiproliferative effect of scFv43M2. The proliferation of HPV16-positive cells was hindered also when they were co-cultured in transwells with cells producing EVs uploading the Nefmut/43M2scFv fusion. This result confirmed the ability of therapeutic exosomes to be released and reach other cells, with interesting implications for in vivo translation. The established proof-of-concept that the EV-mediated delivery of scFvs can target intracellular antigens renders it feasible the development of this system for in vivo use. In addition, the possibility to obtain recombinant exosomes from the host following the administration of a genetic construct as a vaccine, suggests a feasible translation to humans of this delivery system for anti-E6 and E7 intrabodies [100]. This would also take advantage of the capacity of the recipient organism to produce the exosomes. Once the technology is optimized, the intramuscular injection of DNA plasmids expressing antibody constructs followed or not by electroporation, will permit the exosome-loaded antibodies to reach several body districts. As the antibodies are specific for the HPV oncoproteins, such broad distribution will not result in off-target effects while potentially affecting any metastatic cells derived from the primary tumour. Of course, further experiments are necessary to clarify the route followed by exosomes loaded with a therapeutic cargo in the recipient organism, and to establish dosages and timing of administration.

### 6.4. Viral Vector-Based Methods

In the last 30 years, several clinical trials used viral vectors for gene transfer. Gamma-retroviral and lentiviral vectors for haematological cancers; adenoviral vectors for prostate, ovarian and bladder cancer; and adenovirus-associated vectors for pathologies other than cancer were employed with more or less success, and are still the object of preclinical and clinical proof-of-concept studies [101]. Therefore, on the basis of the effective antibody expression achievable in vitro through the transduction of tumour cells with recombinant retroviruses [71,85], and of the advanced state of clinical studies, we believe that viral vectors can be considered a valid resource in addition to the non-viral systems for in vivo antibody delivery. Noteworthy, HPV-associated lesions have a confined localization that renders them accessible to topical therapy whatever the delivery system chosen. Furthermore, the expression of the target oncoproteins being limited to cancer cells represents an additional advantage for the safety of a therapy designed to inhibit protein–protein interactions such as that based on recombinant antibodies.

### 6.5. Ultrasound-Based Methods

The Ultrasound-mediated targeted delivery (UMTD) is a non-invasive method that is attracting increasing interest for many biochemical applications including immunotherapy of tumours. UMTD combined with microbubbles allows delivery of therapeutic molecules precisely in the tumour site. In fact, oscillation and cavitation of microbubbles under the influence of the acoustic beam causes the reversible formation of localized pores of about 100 nm in diameter in the cell membrane [102]. This phenomenon, known as sonoporation, allows the passive release of therapeutic molecules into target cells. The feasibility and specificity of sonoporation for anti-16E6 mAb delivery to cervical carcinoma cell lines were assessed in the in vitro study outlined above, although the effect obtained was transient and incomplete as it affected p53 levels but did not induce apoptosis [70]. However, the issue of delivery to nucleus, which probably underlies the observed partial efficacy, could be addressed using smaller antibody formats provided with NLS. Sonoporation is increasingly explored for both passive and active immunotherapy in vivo. For example, dendritic cells (DC) sonoporated with antigen mRNA and immunomodulating TriMix mRNA were successful in inhibiting tumour growth in mice [103]. Ultrasound in combination with microbubbles even allowed the Herceptin mAb (trastuzumab) to cross the blood-brain barrier in mice, thus opening up the possibility of treating brain metastases of breast cancer [104]. However, translation of the methodology to human therapy requires further investigation on the possible elicitation of immune response by microbubbles, the exact mechanism of the therapeutic material release, the size-based microbubble capacity of penetrating cell membranes, and the excretion of microbubbles from the body.

## 7. Conclusions and Perspectives

Currently, recombinant antibodies for targeting antigens involved in the pathogenesis of a variety of diseases are obtainable by robust methodologies of immunization and in vitro screening. Nevertheless, their use as therapeutics may require optimization of crucial characteristics such as binding specificity and affinity, solubility, and pharmacokinetics, as well as setting up an appropriate delivery system. The possibility of designing bs antibodies that combine binding domains from different parental antibodies can expand the binding capacity of a single molecule. Bs antibodies could target at the same time multiple antigens such as E6 and E7, or multiple epitopes on the same antigen (such as DBD and E6AP binding domain on E6) but their solubility and stability may be affected and require corrections [105].

The implementation of therapeutic antibodies is an exciting challenge that can now make use of refined computational methods, allowing to design antibodies with the highest affinity towards antigens of interest [106], to predict the biochemical and biophysical characteristics of specific sequences, and to determine whether they conform well to antibodies that have already reached clinical stage [83]. Given that the global burden of HPV-associated cancers is unacceptably high, major efforts are required for the effective prevention and treatment of these tumours. A therapy for HPV-associated lesions relying on antibodies would present some advantages over more conventional systems of immunization such as, for example, those based on triggering tumour rejection. Specificity is among the main merits, due to the possibility of inhibiting the activity of oncoproteins that are expressed only in tumour cells. A further benefit is that such a therapy, not based on the need to elicit the host immune response, can also be effective in subjects immunosuppressed by natural or induced causes as co-infections or pharmacological treatments. The extraordinary potential of anti-HPV recombinant antibodies makes them key tools in the global strategy of fighting HPV-associated cancers.
